# Comparative Genomics of* H. pylori* and Non-Pylori* Helicobacter* Species to Identify New Regions Associated with Its Pathogenicity and Adaptability

**DOI:** 10.1155/2016/6106029

**Published:** 2016-12-18

**Authors:** De-Min Cao, Qun-Feng Lu, Song-Bo Li, Ju-Ping Wang, Yu-Li Chen, Yan-Qiang Huang, Hong-Kai Bi

**Affiliations:** ^1^Center for Scientific Research, Youjiang Medical University for Nationalities, Baise, Guangxi 533000, China; ^2^School of Medical Laboratory Sciences, Youjiang Medical University for Nationalities, Baise, Guangxi 533000, China; ^3^School of Basic Medical Sciences, Youjiang Medical University for Nationalities, Baise, Guangxi 533000, China; ^4^Department of Pathogenic Biology, School of Basic Medical Sciences, Nanjing Medical University, Nanjing, Jiangsu 211166, China

## Abstract

The genus* Helicobacter* is a group of Gram-negative, helical-shaped pathogens consisting of at least 36 bacterial species.* Helicobacter pylori* (*H. pylori*), infecting more than 50% of the human population, is considered as the major cause of gastritis, peptic ulcer, and gastric cancer. However, the genetic underpinnings of* H. pylori* that are responsible for its large scale epidemic and gastrointestinal environment adaption within human beings remain unclear. Core-pan genome analysis was performed among 75 representative* H. pylori* and 24 non-*pylori Helicobacter* genomes. There were 1173 conserved protein families of* H. pylori* and 673 of all 99* Helicobacter* genus strains. We found 79 genome unique regions, a total of 202,359bp, shared by at least 80% of the* H. pylori* but lacked in non-*pylori Helicobacter* species. The operons, genes, and sRNAs within the* H. pylori* unique regions were considered as potential ones associated with its pathogenicity and adaptability, and the relativity among them has been partially confirmed by functional annotation analysis. However, functions of at least 54 genes and 10 sRNAs were still unclear. Our analysis of protein-protein interaction showed that 30 genes within them may have the cooperation relationship.

## 1. Introduction

*H. pylori* is a Gram-negative, spiral-shaped epsilon-proteobacterium. It colonizes 50% of the world's human population, even as high as 80% in developing countries, making it one of the most successful pathogens [1, 2]. This bacterium can cause gastrointestinal disease, such as gastritis, peptic ulcer disease, gastric adenocarcinoma, and mucosa-associated lymphoid tissue (MALT) lymphoma [3–5]. As research continues, a great number of non-pylori* Helicobacter* species (NPHS) inhabiting in a wide variety of human beings, mammals, and birds have been found [6]. Until now, there are at least 36 species of the* Helicobacter* genus that have been studied (http://www.bacterio.net/helicobacter.html).

The* Helicobacter* genus strains have been detected in more than 142 vertebrate species [7]. Among them,* H. pylori* is the major pathogenic bacterium in human beings. Besides* H. pylori*, some NPHS were also found to associate with human body function disorders [8]. For instance,* H. heilmannii, H. winghamensis, H. pullorum*, and* H. canis* were considered as causative agent of stomach and intestinal diseases [9–11].

Many genome regions of* H. pylori*, involved in the mechanism of pathogenesis and adaption to the host environment, have been identified and studied. The well-known Cag-pathogenicity island, an approximately 40 kb DNA region that encodes type IV secretion system (T4SS) and effector molecule cancer-associated gene toxin (cagA), has been proved to play a significant role in pathogenicity [12, 13]. The urea enzymes encoded by urease gene cluster can catalyze the hydrolysis of urea to ammonium and carbon dioxide. It is an influential colonization factor and contributes to gastric acid resistance [14]. Vacuolating cytotoxin (VacA) is a pore-forming toxin that implicates in altering host cell biology, including autophagy, apoptosis, cell vacuolation, and inhibition of T-cell proliferation [15–17].

In the past two decades, the whole genome of* H. pylori* and NHPS have been widely sequenced, which give us a more open field of version to study its pathogenicity and adaption mechanism. Previous studies indicated that* H. pylori* has a high rate of gene recombination and unusual genetic flexibility, and those traits were considered to be helpful for the adaption to the dynamic environment [18, 19]. Even though massive virulence factors of them have been studied, the mechanisms that the essential genome components of* H. pylori* lead to its large scale epidemic and gastrointestinal environment adaptation within human beings remain to be further elucidated.

In this study, comparative analysis of whole genome was made to reveal general character and characteristics of* Helicobacter* genus [20].* H. pylori* and NHPS genomes that are available on public databases were used in the analysis. We intended to identify potential regions of* H. pylori* genomes that are responsible for its epidemicity and adaptability. In addition, comparative genome analysis among* Helicobacter* genus species can give a comprehensive insight into the genomic diversity in each species and help us to understand the relationship well among them.

## 2. Materials and Methods

### 2.1. Data Selection and Management

*Helicobacter* genus involves at least 36 species, while* H. pylori* is given more prominence for medicine. There are multiple complete genomes of them available on public databases, and the genomic data was acquired from NCBI FTP site (ftp://ftp.ncbi.nlm.nih.gov/genomes/) in this study. 99 genomes were selected, including 75 complete* H. pylori* genomes and 24 NPHS genomes, which belong to 19 species (released at the analysis time). To ensure the accuracy and consistency of initial data, chromosome, plasmids, and scaffolds of each candidate strain were concatenated by sequence “NNNNNCATTCCATTCATTAATTAATTAATGAATGAATGNNNNN” to establish a pseudochromosome for further analysis [21].

In order to get the accordance dataset and avoid contradiction that was caused by difference of the gene prediction method applied in different projects, a single gene finding program, Glimmer version 3.02 [22], was used to predict open reading frames (ORFs). The ORFs were removed while their start or end position was inside the sideward sequence. The predicted results and raw databases information were corroborated to one another. And the program RNAmmer-1.2 [23] was used to predict full length of rRNA gene sequences. The size, GC content, number of genes, source, and other characteristics of all selected genomes were listed in Table 1.

### 2.2. Phylogenetic Analysis of 16S rRNA

In order to better understand the phylogenetic relationships among* Helicobacter* species, a phylogenetic tree was constructed using the 16S rRNA genes obtained from the 99 genomes. In addition,* Campylobacter jejuni* and* Campylobacter fetus* were used as outgroup. Multiple sequence alignment of 101 16S rRNA genes was performed using MAFFT version 7.123b [24]. The phylogenetic tree was inferred by the Neighbor-Joining method [25] using MEGA7 [26]. To estimate the consensus tree, 1000-bootstrap resampling was done.

### 2.3. Cluster Analysis of Core and Pan Genome

Orthologous group analyses were performed with software OrthoMCL version 2.0.9 [27], which could generate a similarity matrix normalized by species representation relationship of sequences, and it was then grouped using the Markov Clustering Algorithm (MCL) [28]. All-against-all BLASTP comparisons were used to get pair sequences of protein dataset in OrthoMCL at start. An *E*-value cutoff of 1*e* − 5 and the aligned sequence length longer than the coverage of 50% of a query sequence was chosen to perform OrthoMCL.

A family matrix, which was generated from the genome pairwise comparison of the gene contents of any two genomes, was visualized. The gene families obtained from the OrthoMCL were used to get core and pan genome datasets. The number of unique genes and gene families for each individual species relative to other 98 genomes was calculated and visualized with bar graph.

### 2.4. Functional Classification of the Core and Accessory Genome

The dataset was combined into three groups: 75* H. pylori* genomes alone, 24 NPHS genomes alone, and all the tested 99* Helicobacter* genomes. For core and accessory genome of three groups, functional annotation and category were analyzed by performing BLASTP program against database Clusters of Orthologous Groups (COGs, 2014 update, https://www.ncbi.nlm.nih.gov/COG/), respectively [29, 30]. The percentage of each function category was illustrated by bar chart. All the heatmap and bar were plotted by R (https://www.r-project.org/).

### 2.5. Unique Regions Analysis of* H. pylori*

Each of the genomes was aligned to* H. pylori* 26695 using BLASTN program. Then, the genome regions shared by at least 80% of the* H. pylori* meanwhile lacked in NHPS were detected by a Perl script. The genomic lengths of unique regions only greater than 200 bp were considered. If the genomic length between each adjacent unique regions is less than 300 bp, it was regarded as a part of unique region. DOOR (Database for prOkaryotic OpeRons) [31] was used to predicate operons of* H. pylori* 26695 genome. Virulence factor database (VFDB) [32], COG database [29], InterProScan [33], and nonredundant (NR) protein database [34] were used to annotate and predict the functions of these genes within the target region. Furthermore, pfam [35], KEGG [36], GO [37], and TrEMBL [38] were used to discover more about the putative function of the hypothetical proteins of them.

Small noncoding RNAs (sRNAs) are ubiquitous regulators existing in all living organisms. They can impact various biological processes via interacting with mRNA targets or binding to regulatory proteins [39, 40]. RNAspace.org (http://RNAspace.org/), which is a comprehensive prediction and annotation tool of ncRNA [41], was used to predict ncRNA of* H. pylori*. Then, the particular ones contained by unique regions of* H. pylori* (URHP) were detected.

The analysis results were virtualized by BLAST ring image generator (BRIG) [42]. Five* H. pylori* strains, 26695, Cuz-20, J99, PeCan4, and SouthAfrica7, were drawn on the inner rings to represent the* H. pylori* species. URHP were drawn on the outer ring and twenty-four NHPS were drawn between them.

### 2.6. Protein-Protein Interaction Network Analysis of URHP Proteins

To better understand the role of URHP proteins in the* H. pylori* adaption and pathogenicity, protein-protein interaction network analysis of URHP proteins was carried out using Search Tool for the Retrieval of Interacting Genes/Proteins (STRING version 10.0) [43]. The STRING database (http://string-db.org/) is a comprehensive database that could provide a strict assessment and integration of protein-protein interactions, including physical as well as functional interrelationships.

## 3. Results and Discussion

### 3.1. Genome Statistics and Features

*H. pylori* was discovered by Warren and Marshall in 1983 and proved to be the pathogen that caused gastritis [44]. Then, the important pathogen strain* H. pylori* 26695 genome was completely sequenced in 1997 [45]. Altogether, ninety-nine genomes were used in this study and listed in Table 1, including 75 complete* H. pylori* genomes and 24 NPHS genomes, and plasmids were identified within 27 genomes (Table 1). The NPHS, which can be classified into 20* Helicobacter* species, includes 11 completed genomes. Average genome size of all strains is 1,689,380 bp, ranging from 1,435,066 bp (*H. pametensis* ATCC 51478) to 2,559,659 bp (*H. bilis* WiWa). The genomes are relatively small and compact compared with other bacteria, which may indicate a specific adaptation for their obligate pathogenic lifestyles [46, 47]. This genus has a low GC content, whose average GC content is 38.91%, ranging from 33.58% (*H. pullorum *MIT 98-5489) to 47.38% (*H. heilmannii* ASB1.4). The average number of protein coding sequences predicted is 1,730, ranging from 1,432 (*H. pametensis* ATCC 51478) to 2,751 (*H. bilis* WiWa).

The hosts of this genus species have great variety. All the* H. pylori* strains and* H. cinaedi, H. fennelliae, H. heilmannii*, and* H. winghamensis* were originally isolated from humans. The natural hosts of* H. canis, H. bizzozeronii, H. Canadensis, H. felis, H. pullorum*, and* H. suis* are mammals or birds, including pig, cat, dog, and geese. At the same time, the above six NHPS were also found to associate with gastric disease in humans [48–51].* H. acinonychis, H. ailurogastricus, H. bilis, H. cetorum, H. hepaticus, H. himalayensis, H. macacae, H. mustelae, H. pametensis*, and* H. typhlonius* were isolated from nonhuman sources only, which had not been reported in human infection before [52–54].

### 3.2. Phylogenetic Analysis of 16S rRNA

**Figure 1:**
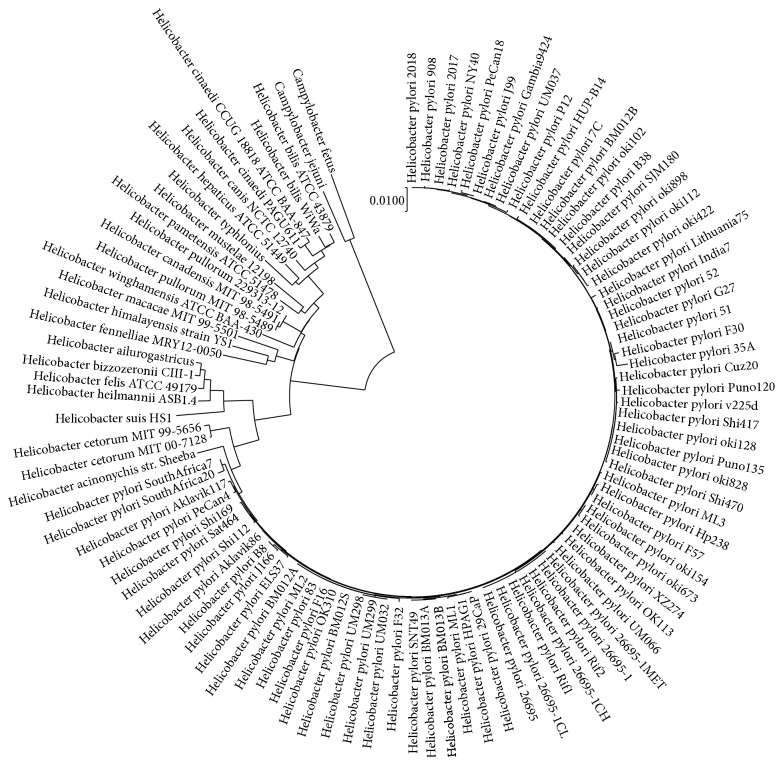
16S rRNA phylogenetic tree of 99* Helicobacter* genus strains and 2* Campylobacter* species was constructed by Neighbor-Joining (NJ) algorithms. The sum of branch length of the optimal tree is 0.47957369. The evolutionary distances were computed using the *p*-distance method.

*Helicobacter* genus species have a wide range of hosts. However,* H. pylori* is one of the most prevalent pathogenic bacteria that comigrated and evolved with human beings all around the world [55]. Each* Helicobacter* species has its own specific or broad hosts or even only survives in several host's organs [56], suggesting that each one of them has developed a balance of adaption with its hosts. In order to better understand the pattern of evolution in this genus, a phylogenetic tree based on 16S rRNA has been constructed for 99* Helicobacter* species with* Campylobacter fetus* and* Campylobacter jejuni* as outgroup. After multiple alignments, the common gaps and missing data were masked. In the final dataset, there were 1,489 bp of each aligned sequence. As shown in Figure 1,* H. acinonychis* and* H. cetorum*, whose nature hosts are cats and aquatic mammals, respectively, are the closest species to* H. pylori*, and* H. pylori* strains have a very close relationship among them.

### 3.3. Homologous Proteome Analysis by Pairwise Comparisons

**Figure 2:**
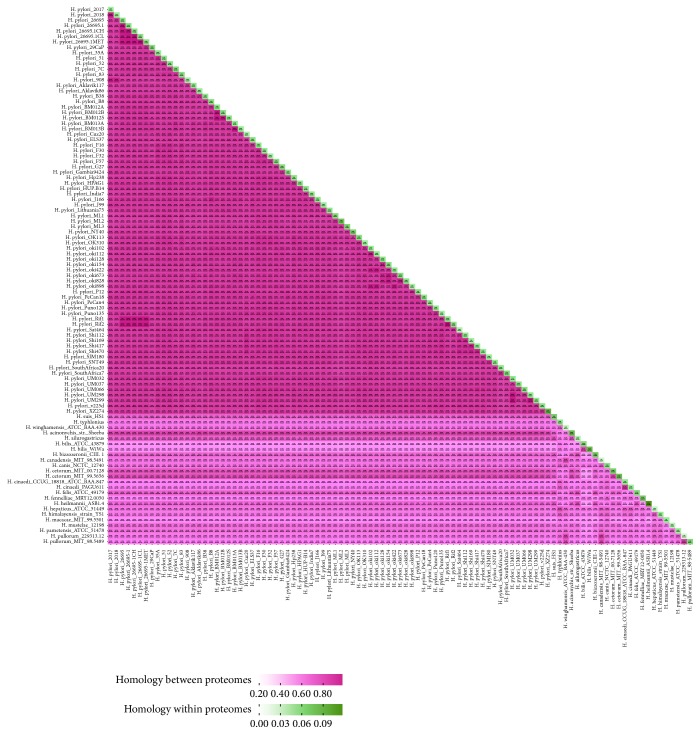
Homologous proteins analysis among proteomes (orthologous) and internal proteomes (paralogous) in the* Helicobacter* genus species. The blocks on the diagonal represent paralogous data and the others represent orthologous data. The percentage of orthologous and paralogous proteins are represented by red and green, respectively. The similarity is indicated by depth of color. The number of homologs and percentage of similarities between/within proteomes are shown in corresponding block.

The whole predicted proteins (proteome) of each strain used in this study were compared to estimate the amount of proteins they shared. The homolog between any two different proteomes ranged from 43.71% (*H. heilmannii* ASB 1.4 versus* H. bilis* ATCC 43879) to 99.87% (*H. pylori* BM013A versus* H. pylori* BM013B), while it is generally to be above 80% within the* H. pylori* strains (Figure 2). The results also showed that* H. acinonychis* (average 81.7%) and* H. cetorum* (average 75.59%) had the highest similarity with* H. pylori*. The relationships shown by the homologous analysis are consistent when compared with the phylogenetic tree. The internal homology against its own proteome ranged from 1.45% (*H. pullorum* 229313-12) to 9.52% (*H. heilmannii* ASB1.4) with average 3.50%, which indicates that this genus's strains have a low redundancy in their genome composition.

### 3.4. Core-Pan Genome Analysis

The core genome, which is responsible for the basic life processes and major phenotypic characteristics, is composed of the gene families that are shared by all the* Helicobacter* species strains. The pan genome is the overall gene families existing in any* Helicobacter* species strain. The pan genome size of 75* H. pylori* genomes is 4,409 with an average of about 39 new gene families extended with followed addition of genome. The increasing speed of pan genome size is almost the same with previous analysis of Ali et al., and their sample size is 39 genomes [57]. For 24 NPHS genomes along, the pan genome size is 12,010, including 4,412 singleton genes. When all NPHS and* H. pylori* genomes were used, the pan genome size was rapidly increased to 14,686, including 8,243 singleton genes. It is more than thrice the size of 75* H. pylori* pan genome size. The above pan genome analytic results suggest that the genomes of* Helicobacter* genus species are open and have diversity. Nevertheless, the core genome size is relatively stable. There are 1,173 gene families shared by all the* H. pylori* genomes, which represent more than 74% of their average gene family contents (~1,565). For all the NPHS genomes along, the core genome size is 682, which is almost the same with the size (673) for all* H. pylori* with NPHS genomes together. It is interesting that there is an obvious difference between the core genome size of* H. pylori* and NPHS. This may indicate that those unique gene families shared by* H. pylori* strains are very relevant to their adaption to unique living environment, pathogenicity, and epidemic.

**Figure 3:**
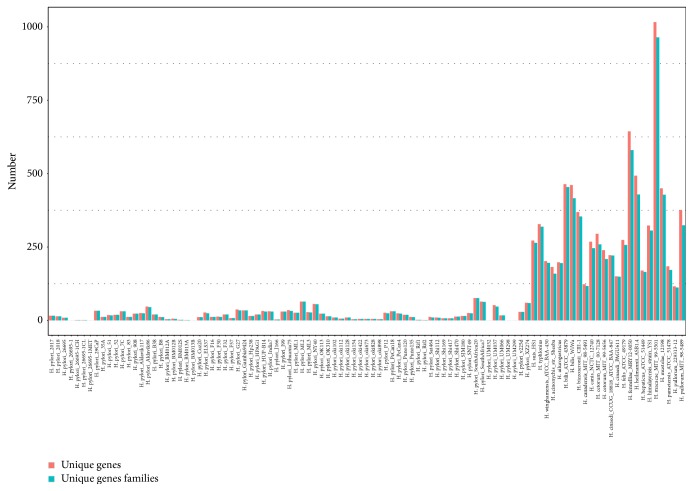
The number of unique genes and gene families for each individual species relative to all 99 genomes. Orange and turquoise bar graphs represent unique genes and gene families for each individual species, respectively.

Estimation of the size of unique genes and gene families for each individual species relative to all 99 genomes was simultaneously carried out (Figure 3).* H. macacae* MIT 99-5501 has the largest number of unique genes and gene families, which are 1,016 and 964, respectively. It accounts for 38.07 percent of its gene contents. The number of unique genes of* H. pylori* is relatively few. This may be due to the fact that too many* H. pylori* genomes were compared with each other. For example,* H. pylori* BM013A genome and* H. pylori* BM013B genome exhibit a high degree of similarity, so only few unique genes exist between them. For all the NHPS, the average number of unique genes and gene families are 325 and 303. It once again implies the obvious genomic plasticity among* Helicobacter* species living in different habits and possessing diverse lifestyles.

### 3.5. COG Category of Core Genome and Accessory Genome

**Figure 4:**
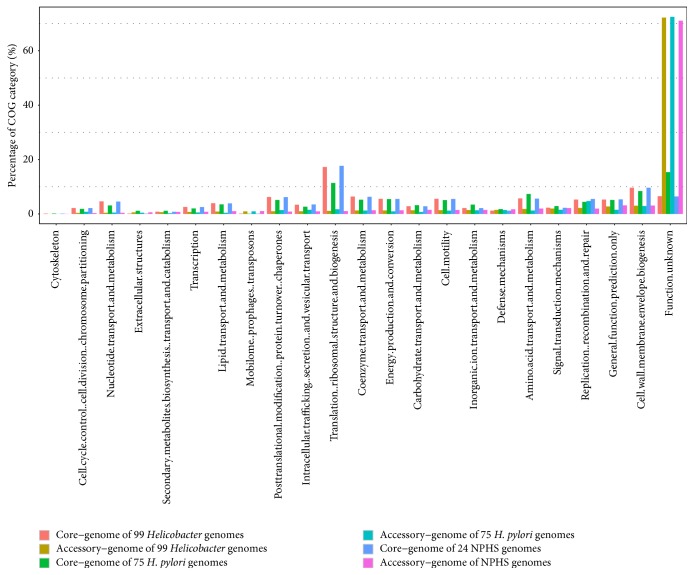
Functional classification of core genome and accessory genome by COG database. Core genome and accessory genome of 99* Helicobacter* genomes and core genome and accessory genome of 75* H. pylori* genomes, along with core genome and accessory genome of 24 NPHS genomes are shown using different colors, respectively.

The core genome and accessory genome of 99* Helicobacter* strains were composed of 673 and 14,013 protein families, separately. For 75* H. pylori* genomes along, the core genome and accessory genome sizes were 1,173 and 3,236, as well as 682 and 11,328 for 24 NPHS genomes along. According to COG category analysis of the above six datasets, possible functions of their gene clusters were identified and subdivided into 23 subcategories. The unassigned gene clusters were put into the same class with function unknown (Figure 4). For three core genome datasets, more than 90% protein clusters were assigned to COG function category. Nevertheless, average 28.1% protein clusters were assigned for three accessory datasets, suggesting that there are still a plenty of proteins without clear biological functions that need to be studied.

In line with what we expected, the significant protein clusters belonging to core genome were assigned to the groups of housekeeping functions. For core genome of 99* Helicobacter* strains, translation, ribosomal structure, biogenesis (category J), and cell wall/membrane/envelope biogenesis (category M) take up 17.26% and 9.65%, respectively, and the percentages are far more than accessory genome. On the contrary, for functional subcategories extracellular structures (category W), mobilome, prophages, transposons (category X), and defense mechanisms (category V), the proportion of accessory genome is greater than core genome. Most of these protein clusters closely related to the interaction of strains and their living environment [58–60]. For instance, type IV pilus (TFP) assembly proteins (category W) are important components of TFP pilus which help* H. pylori* colonization [61]; multiple transposase genes (category X) which can cause antibiotic resistance and transposition are also important to create genetic diversity within species and adaptability to dynamic living conditions [62]; ABC-type multidrug transport system proteins (category V) are used to drug resistance [63] and so on. In addition, the poorly characterized part accounting for more than 70% may be involved in specific adaptations that help* Helicobacter* species survive in novel environments.

### 3.6. Identification of* H. pylori* Unique Regions

**Figure 5:**
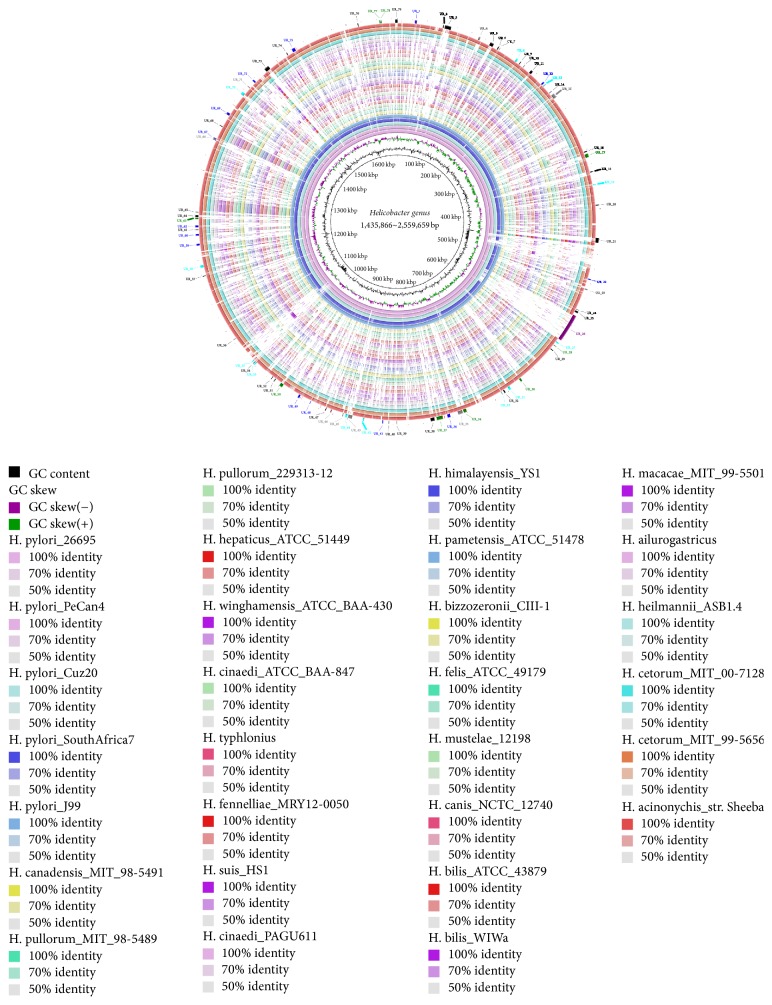
Regions conserved in* H. pylori* and absent from NHPS. From inside to outside, rings 1 and 2 are GC content and GC skew of reference genome* H. pylori* 26695, respectively; rings 3–7 represent* H. pylori* strains while rings 8–31 represent NHPS. The depth of color of rings 3–31 indicates the sequence similarity. Outer ring is the unique regions of* H. pylori* and absent from NHPS. Inside the outer ring, different colors represent different function categories: purple: Cag-PAI; blue: membrane genes; green: transport and metabolism genes; gray: cell growth, division, and basic metabolism; aqua: other functional genes; black: hypothetical genes; red: sRNAs.

A reasonable hypothesis often made in studying bacteria evolution is that the numerous host specific adaptation that a bacterial species displays will be correlated with its specific regions and genes [64]. In this study, seventy-nine sequence segments, total length of 202,359 bp, about 12.4% of the* H. pylori* genome, were identified as unique regions. These regions are shared by* H. pylori* strains but absent from NHPS. The lengths of the unique regions range from 211 bp to 27,269 bp and median length is 1,502. A total of 155 genes are contained in them. Functional annotation of the above genes was performed by VFDB, COG database, InterProScan, and NR database, respectively. Furthermore, the results were integrated (Table S1, in Supplementary Material available online at http://dx.doi.org/10.1155/2016/6106029) and classified into different function categories (Figure 5). Besides, a total of 28 sRNAs within the URHP were identified (Table S2).

In the circular graph, the largest* H. pylori* unique region named UR_26 containing 28 genes can be observed obviously. Average about two genes were contained in each unique region. However, about 82.3% unique regions contain two genes or less. Operons, as the basic units of transcription and cellular functions, have been proved that they are extensively existing in* H. pylori* genome [65]. Within* H. pylori*, sixty unique genes, more than three quarters, are contained in nineteen unique polycistrons. Twenty-three polycistrons are located partly in URHP, in addition to seventy-one monocistrons (Table S1). The known acid induction of* H. pylori* adaptability and virulence operons, such as cag-pathogenicity island, transcriptional regulator (tenA), catalase, and membrane protein (hopT), are included in them [65–67]. These results indicate that* H. pylori* can regulate the expression of those unique genes by control of operons depending on environmental conditions.

A total of 101 genes could get the certain functional annotation within the URHP, compared to the above 4 databases. Unique region UR_26 represents the T4SS, which can deliver effector protein cancer-associated gene toxin (cagA) into gastric epithelial cells. It is reported that T4SS plays a crucial role in the pathogenesis of gastric cancer [12, 60]. Besides T4SS, a plenty of genes, which have been proved strongly to correlate to pathogenicity and adaption, are contained in the unique regions. For instance, membrane proteins babB/hopT, sabB/hopO, and sabA/hopP, and so forth are involved in cell adhesion. These genes facilitate colonization of* H. pylori* and increase immune response, resulting in enhanced mucosal inflammation [68–70]; abundant restriction-modification (RM) system proteins have large effects on gene expression and genome maintenance. They give* H. pylori* the ability to adapt to dynamic environmental conditions during long-term colonization [71]; ABC transporters, MFS transporter, sugar efflux transporter, short-chain fatty acids transporter, and so forth, which are important virulence factors because they play roles in nutrient uptake and secretion of toxins and antimicrobial agents, are important for their interactions with complicated and changeable environments [72–74]. Even though pfam, KEGG, GO, and TrEMBL databases were used for functional annotation, the other 54 genes still cannot get the clear function information, accounting for nearly a third of all URHP genes.

Noncoding small RNAs act as posttranscriptional regulators that fine-tune important physiological processes in pathogens to adapt dynamic, intricate environment [75, 76]. To investigate the regulatory roles of the putative unique sRNAs, we mapped them to the genome of* H. pylori* 26695 [76]. Eighteen of them have matches with genes, unexpectedly (Table S2). Ten sRNAs (SR1, SR2, SR6, SR15, SR20, SR21, SR22, SR23, SR13, and SR25) match perfectly with the known acid induction genes, including eight membrane proteins, DNA polymerase III subunits gamma, tau, and adenine-specific DNA methyltransferase [67, 77]. Besides, SR5 matches with HcpA, which is considered as a virulence factor to trigger the release of a concerted set of cytokines to active the inflammatory response [78]. The small CRISPR RNAs SR7 and SR18 are guides of the CRISPR-Cas system, which was reported as potential participants in bacteria stress responses and virulence [79].

Altogether, it has been proved that the close associations exist between most of the operons, genes, or sRNAs within URHP and adaptability or virulence of* H. pylori*. However, some of them cannot get the certain functional information via current databases, which indicates that our genetic knowledge is still incomplete to explain pathogenicity and adaption mechanism of* H. pylori* fully and these function unknown genes need to be further studied.

### 3.7. Protein-Protein Interaction Network Analysis

**Figure 6:**
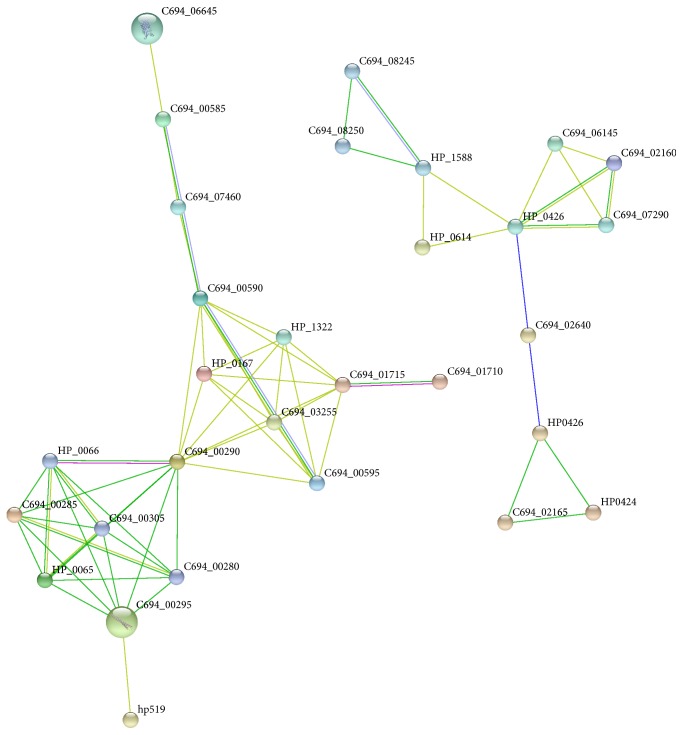
Protein-protein interaction networks of URHP function unknown genes. Thirty proteins are shown in two interaction networks. Network nodes represent proteins and edges represent protein-protein associations. Different colors represent the types of evidence for the interaction.

The 155 URHP genes and 54 genes with unknown functions of* H. pylori* were analyzed using STRING to build protein-protein interaction map, respectively. As shown in Figure S1, a total of 125 genes were assigned into an independent interaction network. It is easy to find two main protein-protein interaction groups: one is well-known cag-pathogenicity island, and the other takes succinyl-CoA-3-ketoacid CoA transferase (encoded by scoA and scoB of operon UO_54), acetone carboxylase (encoded by C694_03570, C694_03590, and C694_03595 of operon UO_55), and acetyl-CoA acetyltransferase (encoded by C694_03555 of operon UO_54) as the center of the interaction map. The second main protein-protein interaction group genes are involved in acetone metabolism. Brahmachary et al. proved that those genes play an important role in survival and colonization of the* H. pylori* in gastric mucosa [80, 81].  Figure 6 shows a possible protein-protein interaction map of the 54 URHP function unknown genes. Thirty proteins were targeted to two divided interaction maps. One includes 18 proteins; the other includes 12 ones. These genes may have synergistic effect on surviving characteristics of* H. pylori*. They could be used as the most possible proteins to further explore the common pathogenic behavior of this pathogen.

## 4. Conclusions

*H. pylori* is an age-old pathogenic microorganism that has infected more than half of the population with strong adaptability. In this study, we presented a comparative genomics analysis of 75 representative* H. pylori* complete genomes and 24 NHPS ones. Pan genome analysis showed that both all* Helicobacter* genus strains and only* H. pylori* species had an open and diverse genome, which may be the result of the different strains that cope with their specific living conditions. However, the core genome is conserved relatively higher. We found 1173 conserved protein families for 75* H. pylori* strains and 673 for all the 99* Helicobacter* genus strains. The regions and genes, which are conserved among* H. pylori* genomes but absent from NHPS genomes, were considered as potential targets that were associated with* H. pylori* pathogenicity and adaptation. Functional annotation of 155 genes within 79 URHP indicated that most of them are well-known pathogenic and adaptive associated ones, such as cag-pathogenicity island, babB, sabB, and ABC transporter, whereas there are still 54 genes of which the biological functions remain unclear. Protein-protein interaction network analysis showed that 30 of them could be assigned to two different interaction networks. Besides, the functional analysis of the operons and sRNAs which were unique to* H. pylori* also showed the intimate association between these genomic structures and its pathogenicity and adaptation. All the URHP, especially those components whose functions remain unclear, could be as potential candidates for further studying and deeply understanding the mechanism of widespread epidemics and pathogenicity in* H. pylori*. In addition, the analysis tools and pipeline used in this study could be as a reference applied to other species.

## Supplementary Material

Supplemental Information includes: Table S1, Unique regions of H. pylori (URHP) and function annotations of relative genes; Table S2, sRNAs shared by all H. pylori but absent from NHPS; Fig. S1 Protein-protein interaction networks of 155 URHP genes.

## Figures and Tables

**Table 1:** *Helicobacter* species genomic information used in the present study.

Organism	Size (bp)	GC (%)	Scaffolds	Plasmids	CDS	rRNA	tRNA	Natural host
*H. acinonychis str. Sheeba*	1557588	38.17	1	1	1706	6	36	Cheetah, tiger
*H. ailurogastricus*	1578404	47.05	9	0	1633	3	36	Feline
*H. bilis ATCC 43879*	2530521	34.7	9	0	2728	3	36	Mice
*H. bilis WiWa*	2559659	34.68	17	0	2751	9	40	Mice
*H. bizzozeronii CIII-1* ^*∗*^	1807534	45.66	1	1	1998	6	36	Dog, cat
*H. canadensis MIT 98-5491* ^*∗*^	1623845	33.69	1	0	1624	9	40	Barnacle, geese, rodent
*H. canis NCTC 12740* ^*∗*^	1932823	44.82	1	0	1914	6	39	Dogs
*H. cetorum MIT 00-7128*	1960111	34.53	1	1	1897	6	38	dolphin, whale
*H. cetorum MIT 99-5656*	1847790	35.54	1	1	1852	6	36	dolphin, whale
*H. cinaedi CCUG 18818 ATCC BAA-847* ^*∗*^	2240130	38.34	1	0	2510	6	39	Human
*H. cinaedi PAGU611* ^*∗*^	2101402	38.55	1	1	2329	6	39	Human
*H. felis ATCC 49179* ^*∗*^	1672681	44.51	1	0	1776	5	36	Cat, dog, rabbit, cheetah
*H. fennelliae MRY12-0050* ^*∗*^	2155647	37.9	49	0	2503	3	38	Human
*H. heilmannii ASB1.4* ^*∗*^	1804601	47.38	1	0	2113	7	41	Human
*H. hepaticus ATCC 51449*	1799146	35.93	1	0	1863	3	37	Mice
*H. himalayensis strain YS1*	1829936	39.89	1	0	1896	6	39	*Marmota himalayana*
*H. macacae MIT 99-5501*	2369528	40.41	4	0	2669	6	39	Macaques
*H. mustelae 12198*	1578097	42.47	1	0	1745	6	38	Ferret
*H. pametensis ATCC 51478*	1435066	40.08	11	0	1432	8	38	Pig, bird
*H. pullorum 229313-12* ^*∗*^	1691799	34.56	60	0	1754	3	36	Poultry
*H. pullorum MIT 98-5489* ^*∗*^	1951667	33.58	44	0	2105	3	36	Poultry
*H. suis HS1* ^*∗*^	1635292	39.91	136	0	1814	5	38	Pig, macaque
*H. typhlonius*	1920832	38.85	1	0	2109	6	39	Mouse
*H. winghamensis ATCC BAA-430* ^*∗*^	1690216	34.74	21	0	1742	3	36	human liver
*H. pylori 2017*	1548238	39.3	1	0	1595	3	36	Human
*H. pylori 2018*	1562832	39.29	1	0	1604	3	36	Human
*H. pylori 26695-1CH*	1667302	38.87	1	0	1667	7	36	Human
*H. pylori 26695-1CL*	1667239	38.87	1	0	1667	7	36	Human
*H. pylori 26695-1*	1667638	38.87	1	0	1667	7	36	Human
*H. pylori 26695-1MET*	1667303	38.87	1	0	1669	7	36	Human
*H. pylori 26695*	1667867	38.87	1	0	1681	7	36	Human
*H. pylori 29CaP*	1667159	38.81	1	0	1704	7	36	Human
*H. pylori 35A*	1566655	38.87	1	0	1583	6	36	Human
*H. pylori 51*	1589954	38.77	1	0	1606	6	36	Human
*H. pylori 52*	1568826	38.94	1	0	1578	6	36	Human
*H. pylori 7C*	1631276	39.01	1	1	1627	7	36	Human
*H. pylori 83*	1617426	38.72	1	0	1634	6	36	Human
*H. pylori 908*	1549666	39.3	1	0	1605	3	36	Human
*H. pylori Aklavik117*	1636125	38.73	1	2	1607	6	36	Human
*H. pylori Aklavik86*	1507930	39.21	1	2	1487	6	36	Human
*H. pylori B38*	1576758	39.16	1	0	1582	7	36	Human
*H. pylori B8*	1680029	38.78	1	1	1673	6	36	Human
*H. pylori BM012A*	1660425	38.88	1	0	1679	7	36	Human
*H. pylori BM012B*	1659060	38.88	1	0	1676	7	36	Human
*H. pylori BM012S*	1660469	38.88	1	0	1683	7	36	Human
*H. pylori BM013A*	1604233	38.96	1	0	1584	7	36	Human
*H. pylori BM013B*	1604212	38.96	1	0	1586	7	36	Human
*H. pylori Cuz20*	1635449	38.86	1	0	1616	6	36	Human
*H. pylori ELS37*	1669876	38.88	1	1	1676	6	36	Human
*H. pylori F16*	1575399	38.88	1	0	1593	6	36	Human
*H. pylori F30*	1579693	38.8	1	1	1582	6	36	Human
*H. pylori F32*	1581461	38.86	1	1	1587	6	36	Human
*H. pylori F57*	1609006	38.73	1	0	1619	6	36	Human
*H. pylori G27*	1663013	38.87	1	1	1672	7	36	Human
*H. pylori Gambia9424*	1712468	39.12	1	1	1694	6	36	Human
*H. pylori Hp238*	1586473	38.7	1	0	1616	5	36	Human
*H. pylori HPAG1*	1605736	39.07	1	1	1595	6	36	Human
*H. pylori HUP-B14*	1607584	39.04	1	1	1597	6	36	Human
*H. pylori India7*	1675918	38.9	1	0	1664	6	36	Human
*H. pylori J166*	1650561	38.93	1	0	1630	6	36	Human
*H. pylori J99*	1643831	39.19	1	0	1629	6	36	Human
*H. pylori Lithuania75*	1640673	38.87	1	1	1659	6	36	Human
*H. pylori ML1*	1629815	38.69	1	0	1701	6	36	Human
*H. pylori ML2*	1562125	38.92	1	0	1764	6	36	Human
*H. pylori ML3*	1635334	38.64	1	1	1744	4	36	Human
*H. pylori NY40*	1696917	38.81	1	0	1751	6	36	Human
*H. pylori OK113*	1616617	38.73	1	0	1649	6	36	Human
*H. pylori OK310*	1595436	38.77	1	1	1595	6	36	Human
*H. pylori oki102*	1633212	38.81	1	0	1630	6	36	Human
*H. pylori oki112*	1637925	38.81	1	0	1635	6	36	Human
*H. pylori oki128*	1553826	38.97	1	0	1565	6	36	Human
*H. pylori oki154*	1599700	38.8	1	0	1626	6	36	Human
*H. pylori oki422*	1634852	38.83	1	0	1641	6	36	Human
*H. pylori oki673*	1595058	38.82	1	0	1623	6	36	Human
*H. pylori oki828*	1600345	38.8	1	0	1618	6	36	Human
*H. pylori oki898*	1634875	38.83	1	0	1612	6	36	Human
*H. pylori P12*	1684038	38.79	1	1	1688	6	36	Human
*H. pylori PeCan18*	1660685	39.02	1	0	1629	6	36	Human
*H. pylori PeCan4*	1638269	38.91	1	1	1622	6	36	Human
*H. pylori Puno120*	1637762	38.9	1	1	1617	6	36	Human
*H. pylori Puno135*	1646139	38.82	1	0	1616	6	36	Human
*H. pylori Rif1*	1667883	38.87	1	0	1678	7	36	Human
*H. pylori Rif2*	1667890	38.87	1	0	1674	7	36	Human
*H. pylori Sat464*	1567570	39.09	1	1	1553	6	36	Human
*H. pylori Shi112*	1663456	38.77	1	0	1651	6	36	Human
*H. pylori Shi169*	1616909	38.86	1	0	1593	6	36	Human
*H. pylori Shi417*	1665719	38.77	1	0	1623	6	36	Human
*H. pylori Shi470*	1608548	38.91	1	0	1612	6	36	Human
*H. pylori SJM180*	1658051	38.9	1	0	1640	6	36	Human
*H. pylori SNT49*	1610830	39	1	1	1599	6	36	Human
*H. pylori SouthAfrica20*	1622903	38.57	1	0	1701	6	36	Human
*H. pylori SouthAfrica7*	1679829	38.42	1	1	1689	6	36	Human
*H. pylori UM032*	1593537	38.82	1	0	1613	6	36	Human
*H. pylori UM037*	1692794	38.89	1	0	1708	6	36	Human
*H. pylori UM066*	1658047	38.62	1	0	1651	6	36	Human
*H. pylori UM298*	1594544	38.82	1	0	1618	6	36	Human
*H. pylori UM299*	1594569	38.82	1	0	1617	6	36	Human
*H. pylori v225d*	1595604	38.94	1	1	1608	6	36	Human
*H. pylori XZ274*	1656544	38.57	1	1	1798	7	36	Human

Note: (1) ^*∗*^NPHS associated with gastric disease in humans.

(2) Latin name, genome size, GC-content, scaffolds number, plasmid number, information of genes, and natural host are listed.

## Abbreviations

MALT: Mucosa-associated lymphoid tissue
NPHS: Non-pylori* Helicobacter* species
T4SS: Type IV secretion system
cagA: Cancer-associated gene toxin
VacA: Vacuolating cytotoxin
ORFs: Open reading frames
COGs: Clusters of Orthologous Groups
VFDB: Virulence factor database
NR: Nonredundant
URHP: Unique regions of* H. pylori*
DOOR: Database for prokaryotic operons
sRNAs: Small noncoding RNAs
BRIG: BLAST ring image generator.
