# Supplementary material for: Comparative Genomics of H. pylori and Non-Pylori Helicobacter Species to Identify New Regions Associated with Its Pathogenicity and Adaptability
Source: Biomed Res Int. 2016 Dec 18;2016:6106029. doi: 10.1155/2016/6106029 (PMC5203880; doi:10.1155/2016/6106029)
Supplement: Supplementary file 1 — Supplemental Information includes: Table S1, Unique regions of H. pylori (URHP) and function annotations of relative genes; Table S2, sRNAs shared by all H. pylori but absent from NHPS; Fig. S1 Protein-protein interaction networks of 155 URHP genes. [file 6106029.f1.pdf]

Supplementary Table S1 Unique regions of *H. pylori* (URHP) and function annotations of relative genes

UR id	UR start	UR end	Operon id	Gene id	gene start	gene end	Strand (+/-)	STRING protein	function
UR_1	23280	25697	UO_1	orf_22	23324	25459	-	hopD	membrane protein (babB/hopT)
UR_2	61346	62217	UO_2	orf_48	61619	61828	+	C694_00270	hypothetical protein
				orf_50	64016	66457	+	C694_00285	hypothetical protein
				orf_51	66454	67023	+	C694_00290	hypothetical protein
				orf_52	67039	67299	+	C694_00295	hypothetical protein
UR_3	63741	72040	UO_3	orf_53	67310	68800	+	C694_00300	5-amino-6-(5-phosphoribosylamino)uracil reductase
				orf_54	68770	69189	+	C694_00305	cell division protein
				orf_55	69191	69544	+	HP_0065	hypothetical protein HP0065
				orf_56	69468	71963	+	HP_0066	ATP-binding protein
UR_4	110620	112829	UO_4	orf_92	110928	112673	-	HP_0104	2', 3'-cyclic nucleotide 2'-phosphodiesterase
			UO_5	orf_106	127931	129118	-	C694_00585	hypothetical protein
UR_5	127589	132369	UO_6	orf_107	129383	130768	-	C694_00590	hypothetical protein
			UO_7	orf_108	131053	132249	-	C694_00595	hypothetical protein
UR_6	139156	140261	UO_8	orf_116	139319	139444	+	HP_0128	hypothetical protein
			UO_9	orf_117	139617	140042	+	C694_00635	hypothetical protein
UR_7	142004	142226	UO_10	orf_119	142028	142129	+	C694_00645	hypothetical protein
UR_8	167642	169944	UO_11	orf_147	168882	169802	+	hcpD	Sel1 repeat protein HcpD
UR_9	174457	175265	UO_12	orf_151	174691	175164	-	HP_0167	hypothetical protein HP0167
UR_10	181448	181798	UO_13	orf_159	181499	181717	-	-	hypothetical protein
UR_11	192313	195290	UO_14	orf_171	193210	194424	-	HP_0186	hypothetical protein
UR_12	214261	216146	UO_15	orf_189	214745	216097	+	C694_01050	membrane protein
UR_13	218066	219071	UO_16	orf_191	218266	219018	-	hcpA	Sel1 repeat protein
UR_14	230507	230997	UO_17	orf_202	230651	230872	+	C694_01120	CopG family transcriptional regulator
			UO_18	orf_206	234973	237048	-	omp5	membrane protein (babB/hopT)
UR_15	234760	240378	UO_19	orf_207	237297	238469	+	HP_0228	sulfate permease
			UO_20	orf_208	238708	240159	-	hopA	membrane protein
UR_16	321866	323095	UO_21	orf_281	321928	322917	+	C694_01535	alginate lyase
				orf_286	325790	326668	+	C694_01560	amidohydrolase
			UO_22	orf_287	326681	327562	+	C694_01565	peptidoglycan deacetylase
UR_17	325693	330332		orf_288	327608	327976	+	C694_01570	hypothetical protein
				orf_289	327976	328941	+	C694_01575	ATP-binding protein
			UO_23	orf_290	328947	330092	-	C694_01580	MFS transporter
UR_18	349703	350646	UO_24	orf_311	349779	350084	+	C694_01710	hypothetical protein
				orf_312	350068	350634	+	C694_01715	hypothetical protein
UR_19	367117	367902	UO_25	orf_327	367133	367885	-	C694_01810	oxidoreductase
UR_20	394336	395240	UO_26	orf_350	394337	395089	-	C694_01950	sporulation protein
				orf_387	439284	441143	-	HP0424	hypothetical protein
UR_21	438354	444565	UO_27	orf_388	441185	442438	-	C694_02165	hypothetical protein
				orf_389	442479	444215	-	HP0426	hypothetical protein HP1409
UR_22	494105	495221	UO_28	orf_424	494379	494939	-	omp11	membrane protein

UR_23	507957	509230	UO_29	orf_434	508122	509066	-	C694_02495	catalase
UR_24	537568	537961	UO_30	orf_456	537802	537918	+	C694_02625	hypothetical protein
UR_25	539660	541958	UO_31	orf_458	539973	541949	+	C694_02640	hypothetical protein
UR_26	546235	583503	UO_32	orf_464	546322	547152	-	hp519	hypothetical protein
			UO_33	orf_465	547328	547675	+	cag1	type IV secretion system protein Cag1
			UO_34	orf_466	548134	549579	+	cag3	type IV secretion system protein Cag3
				orf_467	549589	550098	+	cag4	(virB1) type IV secretion system protein Cag4
			UO_35	orf_468	550217	552463	-	cag5	(virD4) type IV secretion system protein Cag5
				orf_469	552472	553464	-	HP0525	(virB11) type IV secretion system ATPase
				orf_470	553469	554068	-	cag6	type IV secretion system protein Cag6/CagZ
			UO_36	orf_471	554206	559989	-	cag7	(virB10) type IV secretion system protein Cag7/CagY
				orf_472	560004	561572	-	cag8	(virB9) type IV secretion system protein Cag8/CagX
				orf_473	561625	563232	-	cag9	(virB6) type IV secretion system protein Cag9/CagW
				orf_474	563237	563995	-	cagV	(virB8) type IV secretion system protein Cag10/CagV
			UO_37	orf_475	564381	565037	+	cagU	type IV secretion system protein Cag11/CagU
				orf_476	565073	565915	+	cagT	(virB7) type IV secretion system protein Cag12/CagT
			UO_38	orf_477	566126	566716	-	cagS	type IV secretion system protein Cag13/CagS
			UO_39	orf_478	567142	567522	-	cagQ	type IV secretion system protein Cag14/CagQ
			UO_40	orf_479	567955	568299	-	cagP	type IV secretion system protein Cag15/CagP
			UO_41	orf_480	568723	569853	+	cagM	type IV secretion system protein Cag16/CagM
				orf_481	569868	570788	+	cagN	type IV secretion system protein Cag17/CagN
			UO_42	orf_482	570870	571583	-	cagL	(virB5) type IV secretion system protein Cag18/CagL
				orf_483	571580	572725	-	cagI	type IV secretion system protein Cag19/CagI
				orf_484	572736	573848	-	cagH	type IV secretion system protein Cag20/CagH
				orf_485	573864	574292	-	cagG	type IV secretion system protein Cag21/CagG
				orf_486	574347	575153	-	cagF	type IV secretion system protein Cag22/CagF
				orf_487	575155	578106	-	cagE	(virB4) type IV secretion system protein Cag23/CagE
				orf_488	578115	578738	-	cagD	type IV secretion system protein Cag24/CagD
				orf_489	578740	579087	-	cagC	(virB2) type IV secretion system protein Cag25/CagC
			UO_43	orf_490	579231	579458	-	-	cag pathogenicity island protein
			UO_44	orf_491	579921	583481	+	0547	T4SS translocated effector CagA
UR_27	588026	589445	UO_45	orf_496	588072	588755	+	C694_02860	RNA methyltransferase
UR_28	593759	594325	UO_46	orf_502	594037	594273	-	acpP	acyl carrier protein
UR_29	600175	601079	UO_47	orf_510	600177	600944	-	C694_02930	hypothetical protein HP0568
UR_30	656821	659066	UO_48	orf_551	657634	658335	-	C694_03170	ABC transporter ATP-binding protein
			UO_49	orf_552	658630	658965	-	HP_0614	hypothetical protein HP0614
UR_31	675021	677810	UO_50	orf_564	675124	677169	+	C694_03255	hypothetical protein
			UO_51	orf_565	677214	677798	-	C694_03260	NADPH quinone reductase MdaB
UR_32	683582	685165	UO_52	orf_571	684146	684598	-	C694_03295	hypothetical protein
UR_33	687838	688693	UO_53	orf_576	687928	688581	+	frxA	NAD(P)H nitroreductase
UR_34	740425	744508	UO_54	orf_613	740559	741734	+	C694_03555	acetyl-CoA acetyltransferase
				orf_614	741745	742443	+	scoA	succinyl-CoA--3-ketoacid CoA transferase subunit A
				orf_615	742440	743063	+	scoB	succinyl-CoA--3-ketoacid CoA transferase subunit B
				orf_616	743083	744447	+	C694_03570	short-chain fatty acids transporter

				orf_617	745801	747942	+	C694_03590	acetone carboxylase subunit beta
UR_35	745761	752123	UO_55	orf_618	747954	750251	+	C694_03595	acetone carboxylase subunit alpha
				orf_619	750262	750768	+	HP_0697	acetone carboxylase subunit gamma
			UO_56	orf_620	751085	752113	+	C694_03605	hypothetical protein HP0699
UR_36	762802	766591	UO_57	orf_629	762809	763711	+	C694_03650	acetolactate synthase
			UO_58	orf_630	763982	765964	+	homA	membrane protein
			UO_59	orf_637	773625	774083	+	C694_03710	zinc ABC transporter substrate-binding protein
			UO_60	orf_638	774515	776341	+	C694_03715	membrane protein
UR_37	772934	781183	UO_61	orf_639	776481	777473	-	ansA	L-asparaginase II
			UO_62	orf_640	777602	778933	+	dcuA	anaerobic C4-dicarboxylate transporter
			UO_63	orf_641	779008	780897	-	sabA	(sabA/hopP) outer membrane protein
			UO_64	orf_645	784203	785270	-	C694_03750	ATP-binding protein
UR_38	784163	789374		orf_646	785279	785584	-	C694_03755	hypothetical protein
			UO_65	orf_647	785676	787397	-	C694_03760	hypothetical protein
UR_39	834493	835031	UO_66	orf_691	834559	834831	+	0780	hypothetical protein HP0780
UR_40	845203	845413	UO_67	orf_699	845257	845403	-	C694_04045	hypothetical protein
UR_41	853068	853974	UO_68	orf_706	853092	853928	-	C694_04080	membrane protein
UR_42	878299	879962	UO_69	orf_737	878363	879184	+	hpgalT	glycosyltransferase
			UO_70	orf_738	879390	879638	-	C694_04235	ss-DNA binding protein 12RNP2 precursor
				orf_752	894519	895178	-	thiE	thiamine-phosphate synthase
UR_43	894502	899845	UO_71	orf_753	895171	895983	-	thiD	hydroxymethylpyrimidine/phosphomethylpyrimidine kinase
				orf_754	895977	896798	-	thiM	hydroxyethylthiazole kinase
				orf_755	896843	899443	-	C694_04335	type I restriction enzyme R protein (hsdR)
UR_44	901058	903549	UO_72	orf_758	901118	902701	-	HP_0850	type I restriction enzyme M protein (hsdM)
UR_45	906510	907520	UO_73	orf_762	906519	907502	+	guaC	guanosine 5'-monophosphate oxidoreductase
UR_46	922637	924232	UO_74	orf_779	922781	923515	-	cdh	CDP-diacylglycerol pyrophosphatase
			UO_75	orf_780	923867	924196	+	phnA	alkylphosphonate uptake protein (phnA)
UR_47	930256	931124	UO_76	orf_786	930391	930564	+	C694_04495	hypothetical protein
				orf_787	930515	931123	+	HP_0879	hypothetical protein HP0879
UR_48	952836	953370	UO_77	orf_808	952928	953053	-	C694_04625	membrane protein
				orf_809	953064	953363	-	0902	cupin
UR_49	968878	971027	UO_78	orf_820	969238	970782	-	C694_04705	membrane protein
			UO_79	orf_837	995544	996029	+	C694_04810	hypothetical protein
UR_50	995540	999607	UO_80	orf_838	996532	997701	-	C694_04820	proline/betaine transporter ProP
			UO_81	orf_839	999197	999544	-	C694_04830	hypothetical protein
UR_51	1008122	1008877	UO_82	orf_848	1008470	1008832	+	HP_0947	hypothetical protein
UR_52	1012910	1013558	UO_83	orf_854	1012922	1013488	-	C694_04910	hypothetical protein
UR_53	1042108	1043499	UO_84	orf_879	1042237	1043394	+	ftsZ	cell division protein FtsZ
UR_54	1044552	1045073	UO_85	orf_880	1044552	1044854	+	C694_05050	hypothetical protein
UR_55	1047036	1047925	UO_86	orf_882	1047054	1047878	+	pz19b	mechanosensitive ion channel protein
UR_56	1085029	1085310	UO_87	orf_910	1085083	1085244	+	-	hypothetical protein HP1021a
UR_57	1177664	1178407	UO_88	orf_1002	1177684	1178370	-	C694_05750	hypothetical protein
UR_58	1189558	1192654	UO_89	orf_1013	1190157	1190696	-	excC	peptidoglycan-binding protein
				orf_1014	1190763	1192016	-	tolB	protein TolB

UR_59	1219101	1221392	UO_90	orf_1039	1219226	1221316	+	C694_05970	membrane protein
UR_60	1232283	1234285	UO_91	orf_1050	1232603	1234018	+	C694_06025	membrane protein
UR_61	1239535	1240304	UO_92	orf_1056	1239650	1240201	+	C694_06060	hypothetical protein
UR_62	1243302	1245610	UO_93	orf_1059	1243583	1245508	-	hopQ	membrane protein
UR_63	1254165	1255748	UO_94	orf_1067	1254325	1255500	-	sotB	sugar efflux transporter
UR_64	1256712	1259388	UO_95	orf_1069	1256746	1257903	-	C694_06145	hypothetical protein
UR_65	1263078	1263636	UO_96	orf_1073	1263276	1263557	-	C694_06170	flagellar protein
UR_66	1362437	1364093	UO_97	orf_1162	1362519	1363067	+	C694_06645	hypothetical protein (fliZ)
			UO_98	orf_1163	1363131	1363784	-	tenA	Transcriptional regulator (tenA)
UR_67	1364473	1365338	UO_99	orf_1165	1364730	1365215	+	C694_06660	membrane protein
UR_68	1382657	1383359	UO_100	orf_1198	1382720	1383325	+	HP_1322	hypothetical protein HP1322
UR_69	1400125	1403022	UO_101	orf_1218	1400936	1403011	+	HP0227	membrane protein (babB/hopT)
UR_70	1432753	1436250	UO_102	orf_1245	1433295	1436201	+	C694_07075	helicase
UR_71	1445984	1446846	UO_103	orf_1256	1446085	1446477	-	HP_1382	endonuclease
UR_72	1455905	1457772	UO_104	orf_1270	1456007	1456735	+	C694_07210	membrane protein
				orf_1280	1477927	1479663	+	HP_0426	hypothetical protein HP1409
UR_73	1477577	1483763	UO_105	orf_1281	1479704	1480888	+	C694_07290	hypothetical protein
				orf_1282	1481000	1482859	+	HP0424	hypothetical protein
UR_74	1513142	1513961	UO_107	orf_1307	1513143	1513922	-	C694_07460	hypothetical protein
UR_75	1521687	1525911	UO_108	orf_1320	1521892	1524132	+	C694_07525	membrane protein
			UO_109	orf_1321	1524949	1525860	-	C694_07530	hypothetical protein
UR_76	1614876	1615862	UO_110	orf_1397	1615030	1615716	+	C694_07965	hypothetical protein
UR_77	1643914	1645222	UO_111	orf_1422	1643982	1644983	-	C694_08095	iron ABC transporter substrate-binding protein
UR_78	1645802	1646775	UO_112	orf_1424	1645960	1646775	+	HP_1564	ABC transporter substrate-binding protein
			UO_113	orf_1446	1666029	1666790	-	HP_1588	hypothetical protein
UR_79	1664738	1667839	UO_114	orf_1447	1667057	1667680	-	C694_08245	hypothetical protein
				orf_1448	1667681	1667800	-	C694_08250	hypothetical protein

Note: 1. The position information is based on genome of *H. pylori* 26695. STRING protein is the correspond labels used for the protein interaction nodes in Figure 6 and Figure S1. Functional information was annotated by VFDB, COG, InterProScane and NR databases.

2. UR id, UR start, UR end, Operon id, Gene id, gene start, gene end, Strand (+/-), STRING protein id and gene function are listed.

Supplementary Table S2 sRNAs shared by all *H. pylori* but absent from NHPS

ID	Start	End	Strand	Type	Matching gene	Tools
SR1	5,506	5,548	-	SAM	membrane protein (HP0009)	Darn
SR2	24,212	24,278	.	ncRNA	membrane protein (HP0025)	atypicalGC
SR3	25,346	25,409	.	ncRNA	-	BLAST/CG-seq/caRNAc
SR4	64,875	64,962	+	snoR11	-	INFERNAL
SR5	218,832	218,897	+	ncRNA	beta-lactamase HcpA	INFERNAL
SR6	236,700	236,804	.	ncRNA	membrane protein (HP1342)	atypicalGC
SR7	635,392	635,427	-	CRISPR RNA direct repeat element	-	ERPIN
SR8	688,568	688,674	-	snR87	-	INFERNAL
SR9	740,862	740,926	.	ncRNA	Acetyl CoA acetyltransferase	atypicalGC

SR10	742,045	742,119	.	ncRNA	3-ketoacid-CoA transferase subunit A	atypicalGC
SR11	746,847	746,905	.	ncRNA	Acetone carboxylase subunit beta	atypicalGC
SR12	763,284	763,344	.	ncRNA	S-adenosyl-l-methionine hydroxide adenosyltransferase	atypicalGC
SR13	770,646	771,019	.	ncRNA	DNA polymerase III subunits gamma and tau	BLAST/CG-seq/caRNAc
SR14	773,028	773,176	.	ncRNA	-	BLAST/CG-seq/caRNAc
SR15	775,868	775,969	.	ncRNA	membrane_protein	BLAST/CG-seq/caRNAc
SR16	895,871	895,924	.	ncRNA	phosphomethylpyrimidine kinase	atypicalGC
SR17	923,019	923,101	-	sroD	CDP-diacylglycerol pyrophosphatase	INFERNAL
SR18	930,481	930,491	+	CRISPR RNA direct repeat element	-	ERPIN
SR19	938,532	938,603	.	ncRNA	Vacuolating cytotoxin autotransporter	atypicalGC
SR20	965,481	965,594	.	ncRNA	membrane protein (HP0912)	atypicalGC
SR21	967,499	967,550	.	ncRNA	membrane protein (HP0913)	atypicalGC
SR22	1,319,663	1,319,790	.	ncRNA	membrane protein (HP1243)	atypicalGC
SR23	1,401,180	1,401,284	.	ncRNA	membrane protein (HP1342)	atypicalGC
SR24	1,415,226	1,415,331	+	SNORA62	-	INFERNAL
SR25	1,433,041	1,433,098	+	sR16	adenine-specific DNA methyltransferase	INFERNAL
SR26	1,497,079	1,497,140	.	ncRNA	-	atypicalGC
SR27	1,525,762	1,525,812	+	SAM	-	Darn
SR28	1,527,694	1,527,769	+	snoR4	-	INFERNAL

Note: 1. The position information is based on genome of *H. pylori* 26695.

2. sRNAs ID, Start, End, Strand, sRNAs family type, Matching gene and Tools are listed.

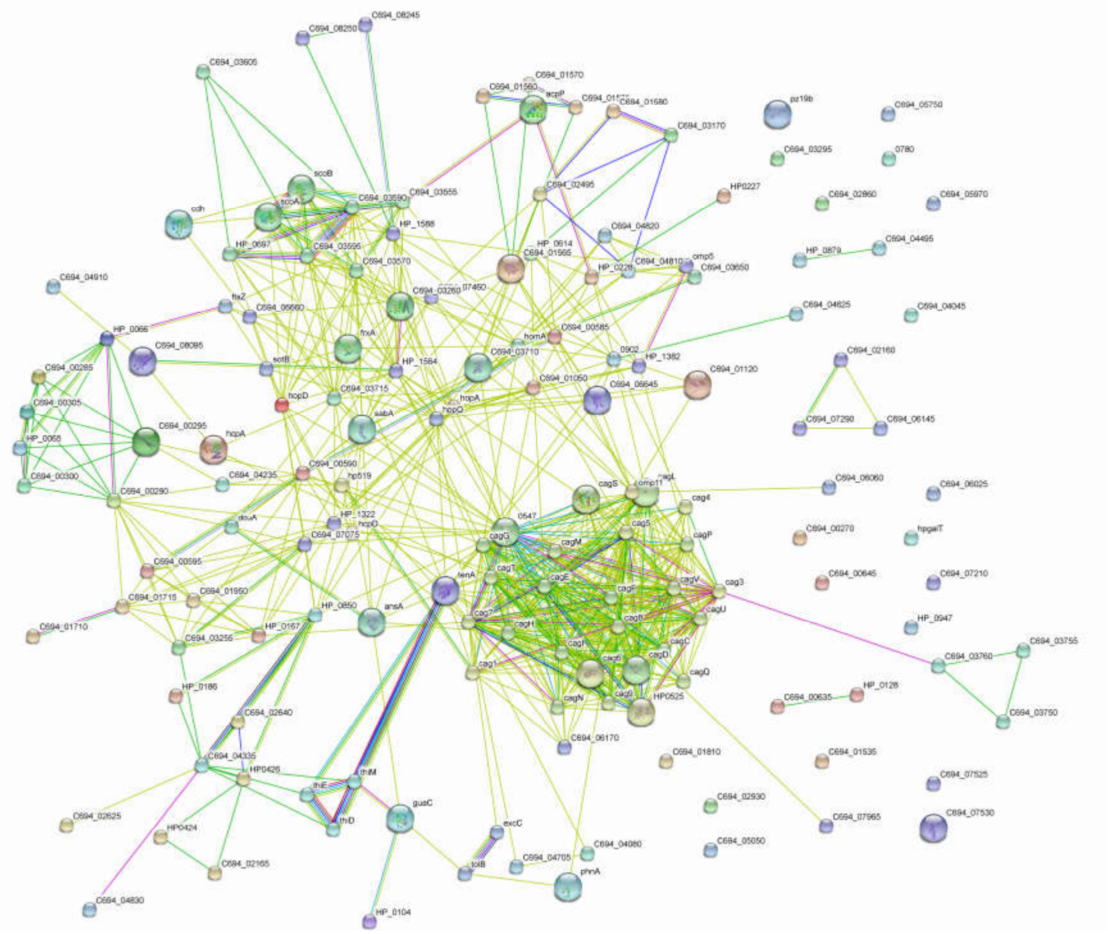

Supplementary Fig. S1 Protein-protein interaction networks of 155 URHP genes. A total of 125 proteins were assigned to one interaction network. Network nodes represent proteins and edges represent protein-protein associations. Different colors represent the types of evidence for the interwork.
